# Supplementary material for: Functional Identification and Genetic Transformation of the Ammonium Transporter PtrAMT1;6 in Populus
Source: Int J Mol Sci. 2023 May 10;24(10):8511. doi: 10.3390/ijms24108511 (PMC10218248; doi:10.3390/ijms24108511)
Supplement: Supplementary file 1 [file ijms-24-08511-s001.zip › Table S1 and Figure S1.pdf]

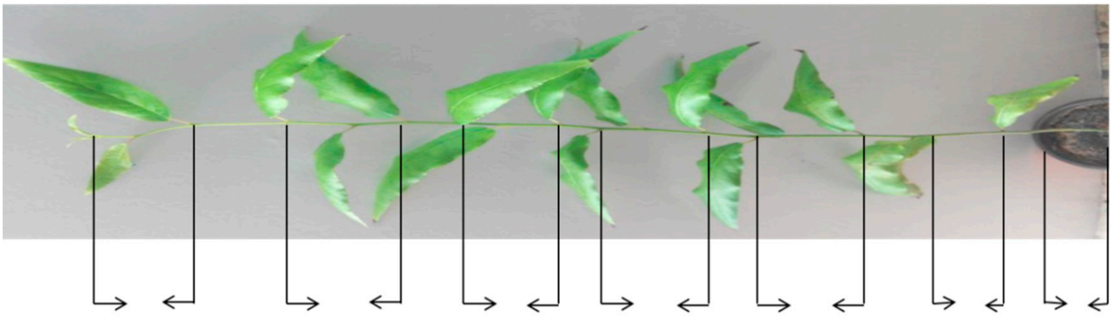

Figure S1. Quantitative sampling of whole-tissue expression patterns of *P.trichocarpa*.

Table S1. Primers used for quantitative RT-PCR (qRT-PCR) and gene cloning.

Primer	Primer Sequence	Enzyme cut site
pEASY-S	ATGGAGGTCTCATGGGA	
pEASY-A	TCATGAATGATTTCGAC	
pYES2-S	CAGAGCTCATTATGGAGGTCTCATGGG	*Sac* I
pYES2-A	CATCTAGATCATGAATGATTTCGACCTTG	*Xba* I
AMT1;6P-S	CACCCGGGTTTTGCACTGCAGT	*Sma* I
AMT1;6P-A	GTAGATCTACCATTCGTATCGTAGGAGAGA	*Bgl* II
pROKE2-S	CAGGATCCATGGAGGTCTCATGGGA	*BamH* I
pROKE2-A	CGGGTACCTCATGAATGATTTCGAC	*Kpn* I
ATACT-S	GGTAACATTGTGCTCAGTGGTG	
ATACT-A	CTCGGCCTTGGAGATCCACATC	
16QPCR-S	TGGGCTCAACGTCTTGGCTCT	
16QPCR-A	TCTCACCACTCCCGCCTCACCT	
NPT II-S	ATGATTGAACAAGATGGATTGCACG	
NPT II-A	TCAGAAGAACTCGTCAAGAAGGCG	
PART1-S	GACTCGAGTTCAACAGCATTTCTCCACTG	*Xho* I
PART1-A	GACTCGAGTGAAATTGACTACATATTTCGG	*Xho* I
PART2-S	GTAAGCTTTGAAATTGACTACATATTTCGG	*Hind*III
PART2-A	GATCTAGATTCAACAGCATTTCTCCACTG	*Xba* I
